# Population-Attributable Risk Estimates for Risk Factors Associated with *Campylobacter* Infection, Australia

**DOI:** 10.3201/eid1406.071008

**Published:** 2008-06

**Authors:** Russell J. Stafford, Philip J. Schluter, Andrew J. Wilson, Martyn D. Kirk, Gillian Hall, Leanne Unicomb, the OzFoodNet Working Group

**Affiliations:** * Queensland Health, Brisbane, Queensland, Australia; † University of Queensland, Brisbane; ‡ Auckland University of Technology, Auckland, New Zealand; § OzFoodNet, Canberra, Australian Capital Territory, Australia; ¶ Australian National University, Canberra; # OzFoodNet, Wallsend, New South Wales, Australia; 1 In addition to some of the authors, the OzFoodNet Working Group for this study included Craig Dalton, Tony Merritt, Rosie Ashbolt, Cameron Sault, Joy Gregory, Robert Bell, Rod Givney, Jane Raupach, Barry Combs, Lillian Mwanri, Jennie Musto, Nola Tomaska, Geoff Millard, Mohinder Sarna, Geoff Hogg, Craig Williams, Janet Li, Karin Lalor, Nittita Prasopa-Plazier, Lyn Mueleners, and Ian McKay.

**Keywords:** Campylobacter, risk factors, Australia, case-control studies, epidemiologic methods, research

## Abstract

In 2001–2002, a multicenter, prospective case-control study involving 1,714 participants >5 years of age was conducted in Australia to identify risk factors for *Campylobacter* infection. Adjusted population-attributable risks (PARs) were derived for each independent risk factor contained within the final multivariable logistic regression model. Estimated PARs were combined with adjusted (for the >5 years of age eligibility criterion) notifiable disease surveillance data to estimate annual Australian *Campylobacter* case numbers attributable to each risk factor. Simulated distributions of “credible values” were then generated to model the uncertainty associated with each case number estimate. Among foodborne risk factors, an estimated 50,500 (95% credible interval 10,000–105,500) cases of *Campylobacter* infection in persons >5 years of age could be directly attributed each year to consumption of chicken in Australia. Our statistical technique could be applied more widely to other communicable diseases that are subject to routine surveillance.

## Abstract 2

One-sentence summary: Each year, an estimated 50,500 cases in persons >5 years of age can be directly attributed to consumption of chicken.

Foodborne gastroenteritis is a major public health concern in many countries, including Australia. A recent study estimated that 5.4 million cases (95% credible interval [CrI] 4.0–6.9 million), 15,000 hospitalizations (95% CrI 11,000–18,000), and 80 deaths (95% CrI 40–120) annually are caused by foodborne gastroenteritis in Australia (*1*). Norovirus, enteropathogenic *Escherichia coli*, *Salmonella* spp., and *Campylobacter* spp. accounted for 88% of the estimated 1.5 million (95% CrI 1.0–1.9 million) cases of foodborne disease caused by known pathogens.

Among known foodborne pathogens, *Campylobacter* spp. are the most frequently reported enteric pathogens in Australia (*2*). The incidence of *Campylobacter* infection steadily increased from 1991 through 2001 but has been relatively stable since. In 2005, >15,000 cases were reported in Australia, a crude rate of 113.0/100,000 population. However, because of underreporting, ≈223,000 *Campylobacter* infections are estimated to occur annually; ≈75% of these are foodborne (*3*). Most of these infections are sporadic.

Case-control studies have identified a range of different risk factors for infection; consumption of chicken is the most frequently reported (*4*–*9*). Some of these studies report population-attributable fractions associated with independent risk factors, but no estimates of the total magnitude of infection caused by chicken or other risk factors have yet been reported. Using a multicentered, prospective case-control study, we aimed to develop a multivariable logistic regression model that identified independent foodborne and nonfoodborne risk factors for *Campylobacter* infection for this sample (*7*) and calculate population-attributable risk (PAR) proportions. These PARs were then combined with annual *Campylobacter* infection surveillance data to estimate the total number of infections (with associated CrIs) among persons >5 years of age attributable to specific risk factors that occur in the community each year in Australia.

## Methods

### Study Design and Population

From September 2001 through August 2002, a multicenter, prospective case-control study was conducted across 5 of the 8 states and territories in Australia to identify risk factors for *Campylobacter* infection in persons >5 years of age. These jurisdictions were those with legislation that required physicians and laboratories to notify health departments about patients infected with *Campylobacter*. At the time of this study, the population of the 5 states combined was ≈12 million, and the total population of Australia was ≈19 million.

### Case-Patients and Controls

A case-patient was defined as a person >5 years of age reported with a culture-positive stool result for *Campylobacter* infection and a recent history of acute diarrhea, who was not part of an outbreak investigation unless identified as the index patient. Controls were sourced from a national control bank and frequency matched to case-patients by age groups in each state. The age groups were selected on the basis of potential variation in risk factors due to different behavior at different ages. Age groups were children (5–9 years), adolescents (10–19 years), young adults (20–29 years), middle-aged adults (30–59 years), and elderly (>60 years).

A total of 881 case-patients and 883 controls were recruited for this study. A telephone-administered questionnaire was used to collect detailed information on exposures in the 7 days before onset of illness for case-patients and in the 7 days before interview for controls. The questionnaire comprised several sections, each representing a separate exposure group that listed questions pertaining to potential risk factors related to that group. The following sections were included: meat, poultry and seafood consumption; egg and dairy product consumption; produce consumption; water consumption; food-handling practices; animal and pet exposures; host factors; dining locations outside the home; overseas travel; and demographic information. To measure the effects between illness and consumption of cooked meat products or undercooked meat products, additional information was sought on whether the meat appeared undercooked (pink on the inside) when eaten. A detailed description of the study design, sample, and exposure measurements has been published elsewhere (*7*).

### Data Analysis

A 2-stage model-building strategy was undertaken, first by determining a parsimonious multivariable model for each exposure group, and second by deriving an omnibus parsimonious model that combines significant exposure variables from all the exposure group multivariable models. A more comprehensive description of the analytical model has been published elsewhere (*7*) and is included in the Technical Appendix.

We calculated PARs by using adjusted odds ratios (aORs) from the final multivariable logistic regression model for each variable that was significantly associated with an increased risk for infection, apart from host factors (*10*). Stata statistical software, release 7 (Stata Corp, College Station, TX, USA), was used for calculating 95% confidence intervals (CIs) around the PAR estimates. Using community incidence data derived from adjusted national surveillance data (*3*) coupled with PAR data from our case-control study, we used simulation techniques to estimate the total number of *Campylobacter* infections attributable to specific risk factors that occur in the community each year in Australia and to derive credible regions for these estimates by modeling the uncertainty in each variable component.

### Simulation Methods

We assumed that 223,000 (95% CrI 94,000–363,000) cases of campylobacteriosis occur in Australia in a typical year (*3*). We then adjusted this figure by reviewing Australian notification data for the years 2001 through 2003 (*11*) and applying simulation techniques to estimate the proportion of cases that occur among persons >5 years of age. Similarly, we randomly generated simulated PAR values for each risk factor using aORs from the final model. The simulated campylobacteriosis case numbers and PAR-simulated values were multiplied together to produce distributions of the total number of *Campylobacter* infections attributable to each specific risk factor. Because some distributions are skewed, we present medians and 95% CrIs (defined to be the 2.5 and 97.5 percentiles) for the simulation results. Simulations were undertaken in SAS System for Windows, version 9.1 (SAS Institute Inc., Cary, NC, USA). A detailed description of the simulation technique used to derive these estimates is provided in the Technical Appendix. The full description of the sample and the development of the final multivariable logistic regression model have been published elsewhere (*7*).

## Results

### Multivariable Analysis of Risk Factors

Table 1 reports results of univariable (crude) and multivariable logistic regression analyses for variables within each exposure group (adjusted for state, sex, and education), and the final multivariable model showing frequency and sample size, percentages, and crude odds ratios (ORs) and aORs, together with 95% CIs. The independent risk factors that were identified in the final model explained only a limited proportion of illness (Nagelkerke R^2^ = 0.16). Consumption of undercooked chicken (aOR 4.7, 95% CI 2.6–8.4), consumption of offal (aOR 2.0, 95% CI 1.0– 4.0), ownership of domestic dogs <6 months of age (aOR 2.1, 95% CI 1.1–4.2), and ownership of domestic chickens <6 months of age (aOR 12.4, 95% CI 2.6–59.3) were the only independent risk factors for infection after adjusting for all other variables in the model. Consumption of cooked chicken was positively but not statistically associated with illness and warranted further consideration (aOR 1.4, 95% CI 1.0–1.9, p = 0.06). Eating fresh fish, eating homemade foods containing raw eggs, eating organically grown fruit and/or vegetables, and eating homegrown fruit were independent factors associated with a statistically significant reduced risk for infection. Eating raw salads or vegetables, as measured by the vegetable index variable, was also associated with a reduced risk for infection. Drinking commercial bottled water, placing barbequed cooked meat back on the same plate used for raw meat, having liver disease, and having any immunosuppressive therapy in the 4-week exposure period were all removed from the final model during the sequential backward elimination procedure. None of the investigated 2-factor interactions was statistically significant. There was no reason to suspect the adequacy of the final multivariable model (Hosmer-Lemeshow goodness-of-fit test, p = 0.98). Additional statistical information, including β-coefficients, standard errors, statistical significance tests, and goodness-of-fit statistics for all multivariable models, is provided in Table 1A, Technical Appendix.

**Table 1:** Results of univariable (crude) and multivariable logistic regression analysis for variables within each exposure group and the final multivariable model, *Campylobacter* infection, Australia, 2001–2002*

Exposure group/variables†	Case-patients, n/N (%)	Controls, n/N (%)	Univariable analysis			Multivariable logistic regression analysis (exposure groups)			Final multivariable model‡	
			OR	95% CI		OR	95% CI		aOR	95% CI
Meat, poultry and seafood						Model 1				
No chicken	110/711 (15.5)	162/808 (20.0)	1.0			1.0			1.0	
Chicken, cooked	528/711 (74.3)	618/808 (76.5)	1.3	1.0–1.7		1.3	1.0–1.8		1.4	1.0– 1.9
Chicken, undercooked	73/711 (10.3)	28/808 (3.5)	3.8	2.3–6.3		4.4	2.6–7.5		4.7	2.6– 8.4
Offal	36/852 (4.2)	16/830 (1.9)	2.2	1.2–4.4		2.1	1.1–3.9		2.0	1.0– 4.0
Fresh fish	256/833 (30.7)	332/827 (40.1)	0.7	0.5–0.8		0.6	0.5–0.8		0.7	0.5– 0.9
Eggs and dairy products						Model 2				
Homemade foods containing raw eggs	40/837 (4.8)	70/822 (8.5)	0.5	0.4–0.8		0.5	0.3–0.7		0.5	0.3– 0.8
Produce						Model 3				
Organic fruit and vegetables	50/805 (6.2)	100/804 (12.4)	0.5	0.3–0.7		0.6	0.4–0.8		0.6	0.4–1.0
Homegrown fruit	84/845 (9.9)	169/828 (20.4)	0.4	0.3–0.6		0.5	0.4–0.7		0.4	0.3–0.6
Vegetable index§										
0 (no vegetables)	141/853 (16.5)	87/830 (10.5)	1.0			1.0			1.0	
1 (1–2)	339/853 (39.7)	305/830 (36.7)	0.7	0.5–0.9		0.7	0.5–1.0		0.7	0.5–1.0
2 (3–4)	352/853 (41.3)	382/830 (46.0)	0.6	0.4–0.8		0.6	0.4–0.9		0.6	0.4–0.9
3 (5–6)	21/853 (2.5)	56/830 (6.7)	0.2	90.1–0.4		0.3	0.1–0.5		0.2	0.1–0.5
Water consumption						Model 4				
Commercial bottled water	72/846 (8.5)	47/820 (5.7)	1.5	1.0–2.3		1.6	1.1–2.3		NS	
Food-handling practices						Model 5				
Barbequed cooked meat placed back on plate used for raw meat	21/511 (4.1)	9/471 (1.9)	2.2	1.0–5.5		2.3	1.0–5.4		NS	
Animal and pet exposure										
Domestic chickens						Model 6				
No domestic chicken	783/846 (92.6)	777/821 (94.6)	1.0			1.0			1.0	
Chicken <6 mo of age	18/846 (2.1)	5/821 (0.6)	3.6	1.3–9.7		5.2	1.5–17.8		12.4	2.6– 59.3
Chicken >6 mo of age	45/846 (5.3)	39/821 (4.8)	1.1	0.7–1.8		1.3	0.8–2.2		1.7	0.9– 3.0
Domestic dogs										
No dog	397/839 (47.3)	452/819 (55.2)	1.0			1.0			1.0	
Dog <6 mo of age	48/839 (5.7)	17/819 (2.1)	3.2	1.8–5.7		2.9	1.6–5.3		2.1	1.1– 4.2
Dog >6 mo of age	394/839 (47.0)	350/819 (42.7)	1.3	1.1–1.6		1.2	1.0–1.5		1.2	0.9–1.5
Host factors						Model 7				
Chronic gastrointestinal condition	101/873 (11.6)	50/831 (6.0)	2.0	1.4–3.0		2.0	1.4–2.9		2.3	1.5–3.4
Liver disease	14/875 (1.6)	2/830 (0.2)	6.7	1.5–61.2		5.1	1.1–23.0		NS	
Any immunosuppressive agent/therapy	35/881 (4.0)	12/833 (1.4)	2.8	1.4–6.0		2.8	1.4–5.5		NS	

*Each model adjusted for state, sex, and education. aOR, adjusted odds ratio; CI, confidence interval; NS, not significant. †The exposure period for foods is 7 d before onset of illness for case-patients and 7 days before interview for controls. ‡After removal of nonsignificant interaction terms. §The vegetable index was created to indirectly measure the range of raw produce consumed in the 7-day exposure period for patients and controls. The values of this index variable represented a count of the number of different types of salad/vegetable foods eaten during the exposure period.

### PAR Proportions

Among the food exposures, the proportion of study patients who reported eating undercooked chicken was 10.3%. The proportion of *Campylobacter* illness in the study population that could be attributed to the consumption of undercooked chicken was estimated to be 8.1% (95% CI 5.2%–11.1%) (Table 2). A further 21.2% (95% CI 0.0%–36.9%) of *Campylobacter* infections in the population could be attributed to cooked chicken. The overall PAR associated with consumption of chicken was 29.3%.

**Table 2:** PAR proportions with 95% CIs and community estimates with 95% CrIs for exposures associated with an increased risk for *Campylobacter* infection in persons >5 y of age, Australia, 2001–2002*†

Risk factor	No. case-patients	Proportion of case-patients (p_i_)	aOR	PAR, %	95% CI	Estimated no. community case-patients	95% CrI
Food exposures							
Chicken consumption							
No chicken	110	0.155	Reference				
Chicken, cooked	528	0.743	1.4	21.2	0.0–36.9	35,500	0–83,500
Chicken, undercooked	73	0.103	4.7	8.1	5.2–11.1	15,000	6,000–26,500
Offal consumption							
No	816	0.958	Reference				
Yes	36	0.042	2.0	2.1	0.0–4.9	3,500	50–8,500
Nonfood exposures							
Dogs (domestic)							
No dog	397	0.473	Reference				
Dog <6 mo of age	48	0.057	2.1	2.9	0.3–4.8	5,000	500–11,500
Dog ≥6 mo of age	394	0.47	1.2	–			
Chickens (domestic)							
No domestic chickens	783	0.926	Reference				
Chickens <6 mo of age	18	0.021	12.4	1.9	0.9–2.9	3,500	1,000–7,000
Chickens ≥6 mo of age	45	0.053	1.7	–			

*PAR, population-attributable risk; CI, confidence interval; CrI, credible interval. †Calculated from adjusted odds ratios (aOR) derived from the final multivariable logistic regression model.

The proportion of campylobacteriosis patients >5 years of age that typically occurs each year in Australia was estimated from the simulations to be 191,000 (95% CrI 79,000–310,000). Applying the simulated PAR estimates to the number of cases of campylobacteriosis in Australia among persons >5 years of age, we estimated 15,000 (95% CrI 6,000–26,500) cases of *Campylobacter* infection could be attributed to eating undercooked chicken in a typical year. Similarly, an additional 35,500 (95% CrI 0–83,500) cases of infection could be attributed to apparently well-cooked chicken. Overall, an estimated 50,500 (95% CrI 10,000–105,500) cases of campylobacteriosis could be attributed to consumption of chicken each year in Australia.

The proportion of case-patients who reported eating offal was 4.2%. The proportion of illness in the study population that could attributed to the consumption of offal was estimated to be 2.1% (95% CI 0.0%–4.9%). This equates to ≈3,500 (95% CrI 50–8,500) cases of campylobacteriosis each year in Australia.

Among the nonfood exposures, ≈5,000 (95% CrI 500–11,500) cases of campylobacteriosis could be attributed to contact with dogs <6 months old each year in Australia. Similarly, an estimated 3,500 (95% CrI 1,000–7,000) cases of campylobacteriosis could be attributed to contact with domestic chickens <6 months old.

## Discussion

The PAR proportions from this study indicate that chicken meat may be associated with >50,000 cases of *Campylobacter* infection each year in Australia. These figures provide a strong argument for government and industry to focus efforts on reducing contamination of chicken carcasses with *Campylobacter* through either improved on-farm control or interventions during processing. In addition, the figures justify the continued need for government to continue educating consumers and foodhandlers about the risks associated with the handling of raw chicken and the potential for cross-contamination in the kitchen.

Several case-control studies of sporadic *Campylobacter* infection have calculated PARs of independent foodborne risk factors (*4*,*5*,*9*,*12*,*13*). In these studies, the PAR percentage associated with chicken meat was 4.9%–31%, compared with 29.3% in our study. However, none of these studies extrapolated their PAR proportions to provide estimates of the total magnitude of infection in their study populations. The use of surveillance data coupled with an understanding of underreporting of illness from the community to surveillance systems allows for this extra important step to quantify the extent of illness caused by specific risk factors (*3*,*14*,*15*).

A recent Australian study indicates that 75% (95% CrI 67%–83%) of cases of *Campylobacter* infection may be due to foodborne transmission (*1*). In our study, the foodborne risk factor with the highest attributable risk was cooked chicken, with an estimated median of 21.2% (95% CrI 0.0%–36.9%); followed by undercooked chicken, with an estimated median of 8.1% (95% CrI 5.2%–11.1%); and offal, with an estimated median of 2.1% (95% CrI 0.0%–4.9%). Although the aOR for cooked chicken is considerably lower than that for undercooked chicken, the high proportion of exposed case-patients (74.3% reported eating cooked chicken) explains the higher PAR. The combined significant foodborne attributable risk estimate found in the study, 31.4% (95% CrI 10.4%–46.8%), is <75%, which suggests that transmission of infection from foodborne vehicles other than chicken is likely to occur.

We interpret the risk associated with cooked chicken as most likely due to the consumption of undercooked chicken that was reported by patients as apparently well cooked or from poor handling during the preparation and cooking of raw chicken. Cross-contamination of cooked or ready-to-eat foods from handling raw chicken and poor food hygiene are considered to be alternative routes of transmission of *Campylobacter* infection (*12*,*16*–*21*). Although no other foods were significantly associated with illness in our study, food-based risk factors implicated from case-control studies conducted outside Australia include eating barbecued red meat or sausages, raw seafood, nonpoultry meat prepared at a restaurant, or pork and drinking unpasteurized milk (*4*,*6*,*8*,*9*,*22*). The Nagelkerke R^2^ value of 16% for the final most parsimonious multivariable model also suggests that a considerable proportion of our case-patients had unexplained risk factors. The difficulty associated with recalling exposures is a major limitation of case-control studies designed to identify multiple potential risk factors. Bias caused by misclassification of reported exposures invariably dampens estimated effect sizes and may partly explain the failure to identify significant associations between some potential risk factors and illness. It is also likely that a proportion of unexplained cases were in persons infected by a variety of foods that had been subject to cross-contamination from raw chicken in the kitchen during preparation (*23*). Because eating chicken meat is a relatively common exposure among both patients and controls, our estimates of effect for cooked and undercooked chicken meat may be underestimates, as will be the derived PAR for chicken meat. However, it is reasonable to assume that at least some of the infections that occur in persons in Australia may be acquired from foods other than chicken or offal.

Two Australian case-control studies, including a study of risk factors among young children, have now identified household puppies and domestic chickens as risk factors for *Campylobacter* infection (*7*,*24*). Among persons >5 years of age, an estimated 8,500 cases of infection could be attributed to these 2 exposures in a typical year; the numbers could be expected to be considerably higher if sporadic infections among children <5 years of age were taken into account. These estimates indicate that a substantial portion of disease is caused by transmission of infection through these routes and provide a timely reminder that public health interventions to reduce this infection in the community should not be directed only at foodborne sources. Although variables associated with a reduced risk for infection did not contribute information to this article, several foods were independently associated with a reduced risk for infection, in particular raw fruit and vegetables. A more detailed discussion on factors associated with a reduced risk for infection in our study is published elsewhere (*7*).

The method used in this study provides an innovative approach to calculate estimates of the total magnitude of infection associated with a specific risk factor in a population, including an estimate of uncertainty. The required components for these calculations include 1) the PAR obtained from a case-control study in which estimates of effect can be generalized to the population under study and 2) an estimate of total community incidence. The method used to derive the incidence used in this study was from reportable disease data from an existing surveillance system and an estimate of underreporting to the surveillance system. Underreporting factors were derived from data on the proportion of case-patients in the community who visit a doctor (P_D_), the proportion of case-patients seen by a doctor who have a stool sample taken (P_S_), the proportion of correctly identified pathogens in stool samples submitted to laboratories (P_L_), and the proportion of positive results that are reported to the surveillance system (P_R_). The product of these proportions (P_D_ × Ps × P_L_ × P_R_) is the reported fraction (*3*). The extent and nature of underreporting will vary with different surveillance systems and for different pathogens. In the future, as more refined methods for calculating the degree of underreporting are developed, these estimates will become more accurate.

PAR estimates are useful for providing a measure of the proportion of illness that can be attributed to individual or multiple causal factors; however, in case-control studies, errors in the estimates of the proportion of cases exposed to a risk factor and/or errors in the estimate of ORs may lead to biased PAR estimates. For example, 1 requirement for estimating PAR is that the study patients be randomly selected from the population of interest and that exposure information be reported without bias. One could argue that the use of culture-confirmed cases in our study is not representative of all *Campylobacter* case-patients in the population because patients with more severe symptoms are more likely to have stools collected and tested (*3*). Therefore, if the exposure information collected from our study patients was different from all case-patients in the general population, the proportion of case-patients exposed to a particular risk factor may be a biased estimate.

Recall and reporting bias are other concerns with case-control studies that may lead to biased estimates of the OR and subsequently the PAR. This is a particular concern for subjective exposures such as undercooked chicken, which are very difficult to measure accurately within a case-control design, so significant associations need to be interpreted with caution. Similarly, it may be difficult for a study participant who reportedly consumed cooked chicken meat to know if the meat was thoroughly cooked. Whether there are differential information biases between case-patients and controls in the reporting of undercooked chicken meat is not clear. In fact, consumption of undercooked chicken may well be systematically underreported by patients. Given the very high prevalence of chicken consumption in the Australian community (81% during the 7-day period before interview among our study controls), finding consumption of undercooked chicken as a risk factor for infection, despite the low reported frequency of exposure, is not surprising. Our PAR estimate for undercooked chicken meat was 8.1%, similar to that reported elsewhere (3%–11%) (*4*,*5*,*9*). No other types of undercooked meat that were measured in our study (e.g., pork, lamb, and beef) were significantly associated with *Campylobacter* infection.

For diseases with multiple risk factors, the PAR estimate for any single factor should be adjusted for possible confounding and interaction by these other factors (*25*,*26*). Multivariable adjustment methods that use logistic regression allow estimates of PAR to a single factor while simultaneously adjusting for other factors in the model. However, if all relevant factors are not included in the model or the model does not have correct parametric form, the adjusted estimates of PAR may be biased (*27*).

The use of simulation techniques provides a simple but robust approach to accommodate asymmetric component distributions and account for uncertainty in our final estimates of the magnitude of foodborne *Campylobacter* infection in the community. Rather than calculate a single point estimate for the number of cases attributable to each foodborne risk factor, a simulated distribution of credible values was generated to model the uncertainty for each component in our calculations. Generating 95% CrIs enabled us to confer a degree of confidence around our estimates.

Intercountry comparison of foodborne disease incidence is difficult without standardization of methods; however, the approach taken in this study may allow those countries that have the available data to conduct similar studies. Furthermore, this model could be adopted or applied more widely to other foodborne and nonfoodborne pathogens under surveillance and enable calculation of population estimates of the magnitude of infection associated with specific risk factors.

## Supplementary Material

Technical Appendix

## References

[1] Hall G, Kirk MD, Becker N, Gregory JE, Unicomb L, Millard G, Estimating foodborne gastroenteritis, Australia. Emerg Infect Dis. 2005;11:1257–64. 16102316 10.3201/eid1108.041367 PMC3320479

[2] Miller M, Roche P, Yohannes K, Spencer J, Bartlett M, Brotherton J, Australia’s notifiable diseases status, 2003 annual report of the National Notifiable Diseases Surveillance System. Commun Dis Intell. 2005;29:1–61. 15966675 10.33321/cdi.2005.29.1

[3] Hall G, Raupach J, Yohannes K. An estimate of under-reporting of foodborne notifiable diseases: *Salmonella, Campylobacter*, Shiga toxin–producing *E. coli* (STEC). NCEPH working paper no. 52. Canberra (Australia): Australian National University; 2006. p. 1–47.

[4] Eberhart-Phillips J, Walker N, Garrett N, Bell D, Sinclair D, Rainger W, Campylobacteriosis in New Zealand: results of a case-control study. J Epidemiol Community Health. 1997;51:686–91. 9519133 10.1136/jech.51.6.686 PMC1060567

[5] Neimann J, Engberg J, Molbak K, Wegener HC. A case-control study of risk factors for sporadic campylobacter infections in Denmark. Epidemiol Infect. 2003;130:353–66. 12825719 10.1017/s0950268803008355 PMC2869971

[6] Kapperud G, Espeland G, Wahl E, Walde A, Herikstad H, Gustavsen S, Factors associated with increased and decreased risk of *Campylobacter* infection: a prospective case-control study in Norway. Am J Epidemiol. 2003;158:234–42. 10.1093/aje/kwg139 12882945

[7] Stafford RJ, Schluter P, Kirk M, Wilson A, Unicomb L, Ashbolt R, A multi-centre prospective case-control study of campylobacter infection in persons aged 5 years and older in Australia. Epidemiol Infect. 2007;135:978–88. 10.1017/S0950268806007576 17134530 PMC2870644

[8] Studahl A, Andersson Y. Risk factors for indigenous campylobacter infection: a Swedish case-control study. Epidemiol Infect. 2000;125:269–75. 10.1017/S0950268899004562 11117949 PMC2869598

[9] Friedman CR, Hoekstra RM, Samuel M, Marcus R, Bender J, Shiferaw B, Risk factors for sporadic *Campylobacter* infection in the United States: a case-control study in FoodNet sites. Clin Infect Dis. 2004;38(Suppl 3):S285–96. 10.1086/381598 15095201

[10] Rockhill B, Newman B, Weinberg C. Use and misuse of population attributable fractions. Am J Public Health. 1998;88:15–9. 9584027 10.2105/ajph.88.1.15 PMC1508384

[11] Australian Government Department of Health and Ageing. National Notifiable Diseases Surveillance System [cited 2005 May 20]. Available from http://www9.health.gov.au/cda/Source/CDA-index.cfm

[12] Evans MR, Ribeiro CD, Salmon RL. Hazards of healthy living: bottled water and salad vegetables as risk factors for *Campylobacter* infection. Emerg Infect Dis. 2003;9:1219–25. 14609455 10.3201/eid0910.020823 PMC3033096

[13] Wingstrand A, Neimann J, Engberg J, Nielsen EM, Gerner-Smidt P, Wegener HC, Fresh chicken as main risk factor for campylobacteriosis, Denmark. Emerg Infect Dis. 2006;12:280–5. 16494755 10.3201/eid1202.050936 PMC3373097

[14] Voetsch AC, Van Gilder TJ, Angulo FJ, Farley MM, Shallow S, Marcus R, FoodNet estimate of the burden of illness caused by nontyphoidal *Salmonella* infections in the United States. Clin Infect Dis. 2004;38(Suppl 3):S127–34. 10.1086/381578 15095181

[15] Wheeler JG, Sethi D, Cowden JM, Wall PG, Rodrigues LC, Tompkins DS, Study of infectious intestinal disease in England: rates in the community, presenting to general practice, and reported to national surveillance. The Infectious Intestinal Disease Study Executive. BMJ. 1999;318:1046–50. 10205103 10.1136/bmj.318.7190.1046 PMC27838

[16] Roels TH, Wickus B, Bostrom HH, Kazmierczak JJ, Nicholson MA, Kurzynski TA, A foodborne outbreak of *Campylobacter jejuni* (O:33) infection associated with tuna salad: a rare strain in an unusual vehicle. Epidemiol Infect. 1998;121:281–7. 10.1017/S0950268898001174 9825778 PMC2809524

[17] Gent RN, Telford DR, Syed Q. An outbreak of campylobacter food poisoning at a university campus. Commun Dis Public Health. 1999;2:39–42. 10462894

[18] Olsen SJ, Hansen GR, Bartlett L, Fitzgerald C, Sonder A, Manjrekar R, An outbreak of *Campylobacter jejuni* infections associated with food handler contamination: the use of pulsed-field gel electrophoresis. J Infect Dis. 2001;183:164–7. 10.1086/317657 11078485

[19] Frost JA, Gillespie IA, O'Brien SJ. Public health implications of campylobacter outbreaks in England and Wales, 1995–9: epidemiological and microbiological investigations. Epidemiol Infect. 2002;128:111–8. 10.1017/S0950268802006799 12002527 PMC2869802

[20] Kirk M, Waddell R, Dalton C, Creaser A, Rose N. A prolonged outbreak of *Campylobacter* infection at a training facility. Commun Dis Intell. 1997;21:57–61. 9090165 10.33321/cdi.1997.21.12

[21] Harris NV, Weiss NS, Nolan CM. The role of poultry and meats in the etiology of *Campylobacter jejuni/coli* enteritis. Am J Public Health. 1986;76:407–11. 3953917 10.2105/ajph.76.4.407 PMC1646506

[22] Kapperud G, Skjerve E, Bean NH, Ostroff SM, Lassen J. Risk factors for sporadic *Campylobacter* infections: results of a case-control study in southeastern Norway. J Clin Microbiol. 1992;30:3117–21. 1452694 10.1128/jcm.30.12.3117-3121.1992 PMC270598

[23] Rodrigues LC, Cowden JM, Wheeler JG, Sethi D, Wall PG, Cumberland P, The study of infectious intestinal disease in England: risk factors for cases of infectious intestinal disease with *Campylobacter jejuni* infection. Epidemiol Infect. 2001;127:185–93. 11693495 10.1017/s0950268801006057 PMC2869737

[24] Tenkate TD, Stafford RJ. Risk factors for campylobacter infection in infants and young children: a matched case-control study. Epidemiol Infect. 2001;127:399–404. 11811871 10.1017/s0950268801006306 PMC2869763

[25] Benichou J. A review of adjusted estimators of attributable risk. Stat Methods Med Res. 2001;10:195–216. 10.1191/096228001680195157 11446148

[26] Whittemore AS. Estimating attributable risk from case-control studies. Am J Epidemiol. 1983;117:76–85. 6823955 10.1093/oxfordjournals.aje.a113518

[27] Coughlin SS, Benichou J, Weed DL. Attributable risk estimation in case-control studies. Epidemiol Rev. 1994;16:51–64. 7925727 10.1093/oxfordjournals.epirev.a036144

