# Supplementary material for: Population-Attributable Risk Estimates for Risk Factors Associated with Campylobacter Infection, Australia
Source: Emerg Infect Dis. 2008 Jun;14(6):895–901. doi: 10.3201/eid1406.071008 (PMC2600281; doi:10.3201/eid1406.071008)
Supplement: Technical Appendix [file 07-1008_Techapp-s1.pdf]

Technical Appendix

METHODS

Development of the multivariable logistic regression model

Initially, separate univariable analyses were performed on all study variables within each exposure group to generate crude odds ratios (OR) with 95% confidence intervals (CI). Exposure variables significantly associated with *Campylobacter* infection in these crude analyses were considered as candidate variables for development of the multivariable model. Using sequential backward elimination of non-significant variables (based on the model deviance statistic), multivariable logistic regression models were then constructed for each exposure group after controlling for state, sex and education. Confounders sex and education were identified from a separate multivariable logistic model of demographic variables and state was considered a design variable (Table 2A). Again using backward elimination, an omnibus multivariable (main-effects) model was then constructed using all significant exposure variables derived from each of the separate multivariable exposure group models as candidate variables and controlling for state, sex and education. Finally, once the most parsimonious multivariable model was identified, two-factor interactions were introduced into the model and backward elimination of non-significant terms were undertaken (based on the model deviance statistic) until the final model was ascertained. The two-factor interactions considered were based on biological plausibility and prior knowledge from the literature. The Hosmer-Lemeshow goodness-of-fit test was performed on all multivariable models to check model adequacy. SPSS (SPSS Version 11.0; SPSS Inc., Chicago) was used for all regression analyses and a significance level of $\alpha = 0.05$ was used to define statistical significance. To reduce the risk of a type I error, only significant variables were included in the multivariable logistic regression models. A detailed description of the analytical approach and pursuant results has been published elsewhere (7).

Simulation methods

To calculate the proportion of campylobacteriosis that occur among persons aged five years and older in Australia, Australian notification data for the years 2001 to 2003 was reviewed (12). The yearly proportions for cases aged 5 years and older among all notified cases reported by the National Notifiable Diseases Surveillance System (NNDSS) between 2001 and 2003 were 84.3%, 85.1% and 87.4% respectively.

Simulations of size 1,000,000 were undertaken in SAS (SAS Institute Inc. *The SAS System for Windows (9.1).* Cary, N.C, USA) to estimate the total number of *Campylobacter* infections attributable to each specific risk factor identified in the final multivariable model using the following steps:

1. *Total Campylobacter case numbers* (N_j). We assumed that 223,000 (95% CrI: 94,000, 363,000) cases of campylobacteriosis occur in Australia in a typical year (3). As this distribution is asymmetrical about its mean, a power transformation of 7/8 was applied (removing the asymmetry in the 95% CrI), 1,000,000 random variates generated, and then these variates were back-transformed to the original scale. We denote these back-transformed variates as N_j for j=1,…,1,000,000.

2. *Eligible Campylobacter case numbers* (n_j). The total campylobacteriosis case numbers variates (N_j) represent expected cases for the whole population and need to be adjusted by the ≥5 years of age eligibility criterion. As the proportion of cases aged 5 years and older in the NNDSS between 2001 and 2003 was strongly time dependent, the simulated total campylobacteriosis case numbers (n_j) were partitioned into three separate one-year periods and eligible (i.e., ≥5 years of age) campylobacteriosis case numbers variates were created by sampling from binomial distributions given by:
   a. $n_j \sim Binomial(p=0.843, N_j)$ for j=1,…, 333,333 (i.e. 2001)
   b. $n_j \sim Binomial(p=0.851, N_j)$ for j=333,3334,…, 666,666 (i.e. 2002)
   c. $n_j \sim Binomial(p=0.874, N_j)$ for j=666,667,…, 1,000,000 (i.e. 2003)

   based on the proportions reported in the NNDSS data (12).

3. *Population attributable risks (PAR) values*. Category-specific attributable risk proportion (PAR_i) was estimated by:

$$PAR_i = \frac{p_i(aOR_i - 1)}{aOR_i} \times 100\%, \qquad \text{(i)}$$

where: aOR_i is the ith category-specific adjusted odds ratio calculated from the logistic regression model and p_i is the proportion of all study cases falling into i^{th} exposure level for a categorical variable with k levels and reference category denoted by $i = 1$. The total population attributable risk proportion (PAR) is given by:

$$PAR = \sum_{i=2}^{k} PAR_i \qquad \text{(ii)}$$

As the distribution of aOR_i is log-normal, PAR_i values for each category level i, i >1, were derived in the following manner. Simulated log(aOR_i) values were randomly generated from a normal distribution with mean and standard deviation estimates derived from the ith exposure category of the risk factor under investigation in the final multivariable logistic regression model, i=2,…,k. These generated log(aOR_i) values were exponentiated, producing aOR_i values. The proportion of people within each of the ith exposure categories, denoted by p_i, was generated from a binomial distribution via: $x_i \sim Binomial(q_i, m)$ where q_i is the proportion of cases in the ith exposure category, m is the number of cases with non-missing data, and $p_i = x_i / m$. Simulated PAR_i and PAR values were then derived by combining the generated aOR_i and p_i given by equations (i) and (ii) above. This process was repeated j=1,…,1,000,000 times.

4. *Attributable Campylobacter case numbers*. Finally, eligible campylobacteriosis case numbers and PAR simulated values derived in Steps 2 and 3 above were multiplied together to produce distributions of the total number of *Campylobacter* infections attributable to each specific risk factor. Because some distributions are skewed, we present medians and 95% credible intervals (defined to be the 2.5 and 97.5 percentiles) for the simulation results.

RESULTS

Table 1A. Results of multivariable logistic regression analysis for variables within each exposure group (adjusted for state, sex and education), and the first model of the multivariable (main effects) model showing Beta-coefficients, Standard Errors (S.E.), Wald Statistics and Hosmer-Lemeshow goodness-of-fit statistics.*

	Multivariable logistic regression analysis (Exposure groups)				First multivariable (main effects) model			
Exposure group / variables	Beta–coefficient	S.E.	Wald statistic	*P* value	Beta–coefficient	S.E.	Wald statistic	*P* value
**1) Meat, poultry and seafood**								
No chicken								
Chicken – cooked	0.27	0.15	3.20	0.07	–0.03	0.26	0.01	0.91
Chicken – undercooked	1.48	0.27	29.55	<0.001	1.55	0.46	11.43	0.001
Offal	0.72	0.33	4.86	0.03	0.66	0.51	1.67	0.20
Fresh fish	–0.44	0.12	13.73	<0.001	–0.54	0.20	7.66	0.006
Hosmer–Lemeshow goodness-of-fit: *P* = 0.70								
2) Eggs and dairy products								
Homemade foods containing raw eggs	–0.72	0.21	11.23	0.001	–0.35	0.37	0.90	0.34
Hosmer–Lemeshow goodness-of-fit: *P* = 0.56								
**3) Produce**								
Organic fruit & vegetables	–0.60		9.41	0.002	–0.33	0.31	1.17	0.28
		0.19						
Home grown fruit	–0.71	0.16	20.88	<0.001	–0.89	0.29	9.50	0.002
Vegetable index:								
0 (no vegetables)								
1 (1–2 vegetables)	–0.36	0.18	4.15	0.04	0.01	0.32	0.002	0.99
2 (3–4 vegetables)	–0.47	0.18	7.27	0.007	–0.26	0.33	0.63	0.43
3 (5–6 vegetables)	–1.37	0.32	18.15	<0.001	–0.84	0.53	2.54	0.11
Hosmer–Lemeshow goodness-of-fit: *P* = 0.48								
**4) Water consumption**								
Commercial bottled water	0.46	0.20	5.15	0.02	0.16	0.30	0.29	0.59
Hosmer–Lemeshow goodness-of-fit: *P* = 0.52								
**5) Food handling practices**								
Barbequed cooked meat placed back on original plate used for raw meat	0.85	0.43	3.84	0.05	0.58	0.54	1.15	0.28
Hosmer–Lemeshow goodness-of-fit: *P* = 0.97								
6) Animal & pet exposure								
Domestic chickens								
No domestic chicken								
Chicken aged <6 months	1.65	0.63	6.82	0.009	2.11	1.17	3.26	0.07
Chicken aged ≥6 months	0.28	0.25	1.21	0.27	0.05	0.51	0.01	0.92
Domestic dogs								
No dog								
Dog aged <6 months	1.08	0.31	12.40	<0.001	0.62	0.50	1.54	0.21
Dog aged ≥6 months	0.22	0.11	4.09	0.04	0.004	0.18	0.00	0.98
Hosmer–Lemeshow goodness-of-fit: *P* = 0.76								

7) Host factors								
Chronic gastrointestinal condition	0.69	0.19	13.42	<0.001	0.94	0.34	7.69	0.006
Liver disease	1.62	0.77	4.42	0.04	–0.35	1.15	0.09	0.76
Any immunosuppressive agent / therapy	1.02	0.35	8.69	0.003	0.05	0.59	0.01	0.93
		Hosmer–Lemeshow goodness-of-fit: *P* = 0.37				Hosmer–Lemeshow goodness-of-fit: *P* = 0.84		

*S.E., Standard Error of coefficient

Table 2A. Results of multivariable logistic regression analysis of all demographic variables together, showing frequency and sample size (n/N), Beta-coefficients, Standard Errors (S.E.), Wald Statistics, Odds Ratios (OR) together with 95% confidence intervals (CI), and the Hosmer-Lemeshow goodness-of-fit statistic.*

Exposure group / variables	Cases n/N	Controls n/N	Beta-coefficient	S.E.	Wald statistic	*P* value	OR	95%CI
**Sex**								
Female	412/881	451/833						
Male	469/881	382/833	0.24	0.11	4.90	0.03	1.3	1.0, 1.6
Age Group (years)								
5–9	66/881	65/833					1.0	
10–19	94/881	94/833	–0.03	0.27	0.01	0.91	1..0	0.6, 1.6
20–29	181/881	144/833	0.07	0.24	0.09	0.77	1.1	0.7, 1.7
30–59	395/881	385/833	–0.12	0.21	0.29	0.59	0.9	0.6, 1.4
60+	145/881	145/833	0.05	0.25	0.04	0.84	1.1	0.6, 1.7
Urban / rural residence								
Urban/town	738/854	695/826						
Rural/remote	116/854	131/826	–0.19	0.16	1.39	0.24	0.8	0.6, 1.1
Education level								
Primary / Secondary to Year 10	89/801	115/775					1.0	
Secondary Years 11&12 or Apprenticeship	290/801	249/775	0.36	0.19	3.71	0.05	1.4	1.0, 2.1
Certificate / Diploma	183/801	176/775	0.25	0.20	1.56	0.21	1.3	0.9, 1.9
University degree	239/801	235/775	0.11	0.21	0.29	0.59	1.1	0.8, 1.7
Household income								
<$25,000	141/708	174/713					1.0	
$25,000–$50,000	206/708	215/713	0.12	0.18	0.46	0.50	1.1	0.8, 1.6
$50,001–$100,000	266/708	236/713	0.29	0.20	2.13	0.14	1.3	0.9, 2.0
>$100,000	95/708	88/713	0.28	0.24	1.36	0.24	1.3	0.8, 2.1
**Health Care / Pensioner Concession card**	279/848	307/824	–0.04	0.15	0.07	0.79	1.0	0.7, 1.3
Indigenous person	19/852	22/824	–0.27	0.36	0.57	0.45	0.8	0.4, 1.6
Season								
Spring	95/881	105/833					1.0	
Summer	153/881	139/833	0.36	0.23	2.35	0.13	1.4	0.9, 2.3
Autumn	320/881	308/833	0.25	0.22	1.25	0.26	1.3	0.8, 2.0
Winter	313/881	281/833	0.37	0.23	2.46	0.12	1.4	0.9, 2.3

State								
Vic	170/881	157/833					1.0	
Qld	196/881	179/833	-0.03	0.19	0.02	0.89	1.0	0.7, 1.4
SA	215/881	182/833	0.14	0.19	0.53	0.47	1.2	0.8, 1.7
WA	134/881	137/833	-0.08	0.19	0.19	0.66	0.9	0.6, 1.3
Tas	166/881	178/833	-0.15	0.19	0.59	0.44	0.9	0.6, 1.3
			Hosmer-Lemeshow goodness-of-fit: *P* = 0.96					

*S.E., Standard Error of coefficient; OR, odds ratio; CI, confidence interval.
